# Phylodynamics of HIV-1 Subtype B among the Men-Having-Sex-with-Men (MSM) Population in Hong Kong

**DOI:** 10.1371/journal.pone.0025286

**Published:** 2011-09-22

**Authors:** Jonathan Hon-Kwan Chen, Ka-Hing Wong, Kenny Chi-Wai Chan, Sabrina Wai-Chi To, Zhiwei Chen, Wing-Cheong Yam

**Affiliations:** 1 Department of Microbiology, Queen Mary Hospital, the University of Hong Kong, Hong Kong Special Administrative Region, China; 2 Integrated Treatment Centre, Special Preventive Programme, Centre of Health Protection, Department of Health, Hong Kong Special Administrative Region, China; 3 AIDS Institute, the University of Hong Kong, Hong Kong Special Administrative Region, China; McGill University, Canada

## Abstract

The men-having-sex-with-men (MSM) population has become one of the major risk groups for HIV-1 infection in the Asia Pacific countries. Hong Kong is located in the centre of Asia and the transmission history of HIV-1 subtype B transmission among MSM remained unclear. The aim of this study was to investigate the transmission dynamics of HIV-1 subtype B virus in the Hong Kong MSM population. Samples of 125 HIV-1 subtype B infected MSM patients were recruited in this study. Through this study, the subtype B epidemic in the Hong Kong MSM population was identified spreading mainly among local Chinese who caught infection locally. On the other hand, HIV-1 subtype B infected Caucasian MSM caught infection mainly outside Hong Kong. The Bayesian phylogenetic analysis also indicated that 3 separate subtype B epidemics with divergence dates in the 1990s had occurred. The first and latest epidemics were comparatively small-scaled; spreading among the local Chinese MSM while sauna-visiting was found to be the major sex partner sourcing reservoir for the first subtype B epidemic. However, the second epidemic was spread in a large-scale among local Chinese MSM with a number of them having sourced their sex partners through the internet. The epidemic virus was estimated to have a divergence date in 1987 and the infected population in Hong Kong had a logistic growth throughout the past 20 years. Our study elucidated the evolutionary and demographic history of HIV-1 subtype B virus in Hong Kong MSM population. The understanding of transmission and growth model of the subtype B epidemic provides more information on the HIV-1 transmission among MSM population in other Asia Pacific high-income countries.

## Introduction

Human Immunodeficiency Virus (HIV) causes a global pandemic at an average rate of 15,000 new infections per day throughout the past three decades. Asia Pacific is a region that is severely suffered from the HIV type 1 (HIV-1) pandemic and over 5 million people in this region are currently living with HIV [1]. HIV-1 subtype B is the predominant genotype in the Western countries while it is also commonly found in most of the developed countries in the Asia Pacific region including Japan, Korea and Australia [2], [3], [4].

Hong Kong is a high-income city of China, which located in the centre of the Asia Pacific region. The first case of HIV-1 infection in Hong Kong was detected in 1984 and the annual number of cases increases continuously in the past three decades [5]. Heterosexual contact was reported to be the major route of HIV-1 local transmission in Hong Kong before 2003 [6]. Afterwards, the escalating number of HIV-1 infections among men-having-sex-with-men (MSM) with prevalence reported as high as 48.7% in 2005 have become a major new concern on public health issue [7]. Recent studies also observed the growing number of subtype B transmission clusters among local MSM starting from 2003 [7], [8]. Internet social network websites and sauna were the major transmission reservoirs of partner sourcing in the local MSM population [9].

The use of bioinformatics analysis on HIV *pol* gene sequence data had elucidated the transmission origin and evolutionary dynamics of HIV epidemics in many Western countries [10], [11]. However, this technology was not widely used in Asian countries for HIV-1 epidemiology and thus limited information about the HIV-1 phylodynamics in Asia was available.

In this study, we aim at investigating the transmission and evolution of HIV-1 subtype B virus in the Hong Kong MSM population. The result of this study was expected to provide more understanding on the epidemic growth model of HIV-1 subtype B virus in the MSM population of developed countries.

## Results

### Study subjects

From the patient epidemiological data, the 125 subtype B infected MSM recruited between 2005 and 2009 had a median age of 34 [Interquartile range (IQR): 28–41] years old, and their median viral load and CD4+ cell count were 28,000 [IQR: 11,000–120,000] copies/mL and 434 [IQR: 315–561] cells/mm^3^ respectively (Table 1). Among the 125 patients, there were 110 (110/125; 88.0%) local Chinese males in which 98 (98/110; 89.1%) of them were infected within Hong Kong. The remaining 12 Chinese males (12/110; 10.9%) caught infection in other countries including the mainland China (5 individuals), Southeast Asia (4 individuals), Europe and the North America (3 individuals). The other 15 patients in the cohort were Caucasian males (15/125; 12.0%). Six of the Caucasian MSM reported to be locally infected in Hong Kong (6/15; 40.0%) while the other 9 individuals (9/15; 60.0%) caught infection outside Hong Kong including the mainland China (2 individuals), Southeast Asia (3 individuals), Australia (1 individual), Europe and the North America (3 individuals).

**Table 1:** Demographic and clinical information for the 125 HIV-1 subtype B infected MSM patients.

	Overall(n = 125)	Cluster 1(n = 45)	Cluster 2(n = 9)	Cluster 3(n = 8)	Others(n = 63)
**Age [median (IQR)], yrs**	34 (28–41)	35 (30–43)	35 (31–39)	34 (27–38)	33 (29–41)
**Ethnic group**					
** Chinese**	110	44	9	7	50
** Caucasian**	15	1	0	1	13
**Median viral load** **(copies/mL)**	28,000(11,000–120,000)	39,000(19,000–120,000)	19,000(9,100–120,000)	23,500(8,250–74,750)	24,000(7,800–115,000)
**Median CD4 cell count** **(cells/mm^3^)**	434 (315–561)	468 (350–581)	399 (359–444)	481 (442–664)	383 (300–511)
**CDC Category**					
** A1-A3**	107	38	9	8	52
** B1-B3**	18	7	0	0	11
**Range of sampling date**	Mar 2005–Apr 2009	Jun 2005–Apr 2009	Jun 2005–Sept 2008	Sept 2006–Jul 2008	Mar 2005–Apr 2009

By using the Stanford HIV drug resistance database, no drug resistance mutation (DRM) was found in any of the samples. However, through the clinical follow-up of these patients, 52 out of 125 individuals (41.6%) would require antiretroviral treatment 6–12 months after the sample collected.

### Bayesian phylogeny of Hong Kong MSM subtype B HIV-1

**Figure 1:**
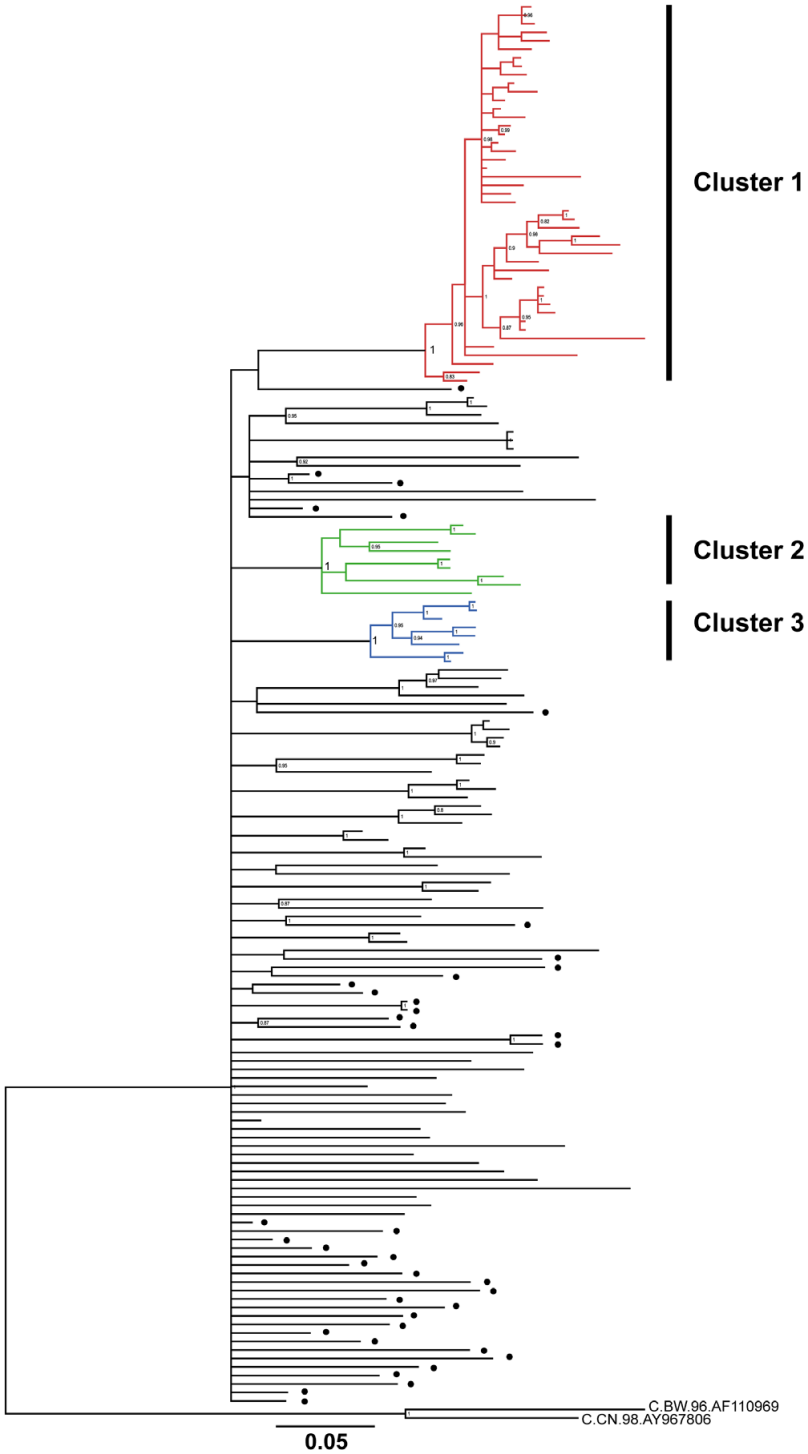
Bayesian phylogenetic tree of 125 subtype B *pol* sequences isolated from MSM in Hong Kong and 40 reference subtype B sequences. Posterior probabilities >0.8 were shown at the nodes of the tree. Solid circle at the right indicate the branches were reference sequences isolated outside Hong Kong. Two HIV-1 subtype C sequences were used as outgroup in the tree. Bar, 0.05 nucleotide subsitution per site.

A Bayesian phylogenetic tree was reconstructed with 165 *pol* sequences, consisting 125 Hong Kong MSM sequences and 40 subtype B reference sequences isolated in Europe and the North America. The tree demonstrated 63 out of 125 Hong Kong sequences (50.4%) were scattered among each other while the other 62 sequences were separated into 3 highly significant transmission clusters (Cluster 1,2 and 3) with posterior probabilities equal to 1 and more than 5 members (Figure 1).

The 63 scattered samples were isolated from 50 Chinese and 13 Caucasians between March 2005 and April 2009. These patients had median age at 33 years old [IQR: 29–41], median viral load at 24,000 [IQR: 7,800–115,000] copies/mL and median CD4+ cell count at 383 [IQR: 300–511] cells/mm^3^ (Table 1). Among these 63 patients, 52 of them were classified as Centers of Disease Control and Prevention (CDC) clinical category A1–A3 with asymptomatic clinical features and another 11 patients were classified as more severe CDC category B1–B3 with the development of clinical symptoms including oral hairy leukoplakia and herpes zoster infection.

Cluster 1 included 45 sequences of 44 Chinese and 1 Caucasian isolated between June 2005 and April 2009. Patients in this cluster had median age at 35 years old [IQR: 30–43], median viral load at 39,000 [IQR: 19,000–120,000] copies/mL and median CD4+ cell count at 468 [IQR: 350–581] cells/mm^3^ (Table 1). Also, 39 patients of this cluster were classified as CDC category A1–A3 and another 6 patients were classified as CDC category B1–B2.

Cluster 2 included 9 sequences of Chinese MSM isolated between June 2005 and September 2008. This cluster had median age at 35 years old [IQR: 31–39], median viral load at 19,000 [IQR: 9,100–120,000] copies/mL and median CD4+ cell count at 399 [IQR: 359–444] cells/mm^3^.

Cluster 3 included 8 sequences of 7 Chinese and 1 Caucasian MSM isolated between September 2006 and July 2008. The median age of this cluster was 34 [IQR: 27–38] years old. The median viral load and CD4+ cell count were 23,500 [IQR: 8,250–74,750] copies/mL and 481 [IQR: 442–664] cells/mm^3^ respectively. All patients in Cluster 2 and 3 did not develop clinical failure symptoms and were all classified as CDC disease category A1–A2.

### Evolutionary rate of HIV-1 subtype B virus among Hong Kong MSM

Bayesian Markov Chain Monte Carlo (MCMC) analyses under a skyline tree prior were used to estimate the time-scale of the subtype B epidemic among the MSM patients in Hong Kong. The mean estimated evolutionary rate (*µ*) for the *pol* gene was 1.94×10^−3^ substitutions site^−1^ year^−1^ under the strict molecular clock model whereas it was estimated at 2.53×10^−3^ substitutions site^−1^ year^−1^ under the relaxed exponential clock model (Table 2). The evolutionary rate of *pol* gene estimated in this study showed no significant difference to the Amsterdam cohort study [11]. To consider the best evolutionary model for this dataset, approximate marginal log likelihoods for the strict and relaxed exponential clock model were calculated. The analysis of Bayes factor showed that the relaxed exponential clock model was strongly supported over the strict clock model for this dataset (2ln BF  = 144.1).

**Table 2:** Population dynamics estimates of the subtype B epidemic among MSM in Hong Kong.

Evolution clock model	Growth model	Rate of evolution(*µ*, site^−1^ year^−1^)	Date of origin (tMRCA)			
			B (MSM-HK)	B (Cluster 1)	B (Cluster 2)	B (Cluster 3)
	Constant	2.01×10^−3^	1979 (1969–1987)	2000 (1997–2002)	1985 (1977–1992)	2000 (1997–2003)
Strict	Exponential	1.82×10^−3^	1975 (1964–1986)	1998 (1995–2001)	1983 (1974–1991)	1999 (1995–2002)
	Logistic	1.63×10^−3^	1977 (1965–1987)	1997 (1993–2001)	1981 (1970–1990)	1998 (1993–2002)
	Skyline	1.94×10^−3^	1982 (1975–1989)	2000 (1997–2002)	1985 (1979–1991)	2000 (1997–2003)
	Constant	2.50×10^−3^	1945 (1875–1988)	1998 (1991–2003)	1989 (1973–2000)	2000 (1993–2005)
Relaxed Exponential	Exponential	2.39×10^−3^	1977 (1960–1990)	1994 (1985–2001)	1989 (1978–1998)	1999 (1993–2004)
	Logistic	2.28×10^−3^	1984 (1974–1993)	1995 (1988–2001)	1989 (1980–1997)	1998 (1992–2003)
	Skyline	2.53×10^−3^	1987 (1978–1994)	1996 (1990–2002)	1991 (1985–1996)	1999 (1994–2004)

**Figure 2:**
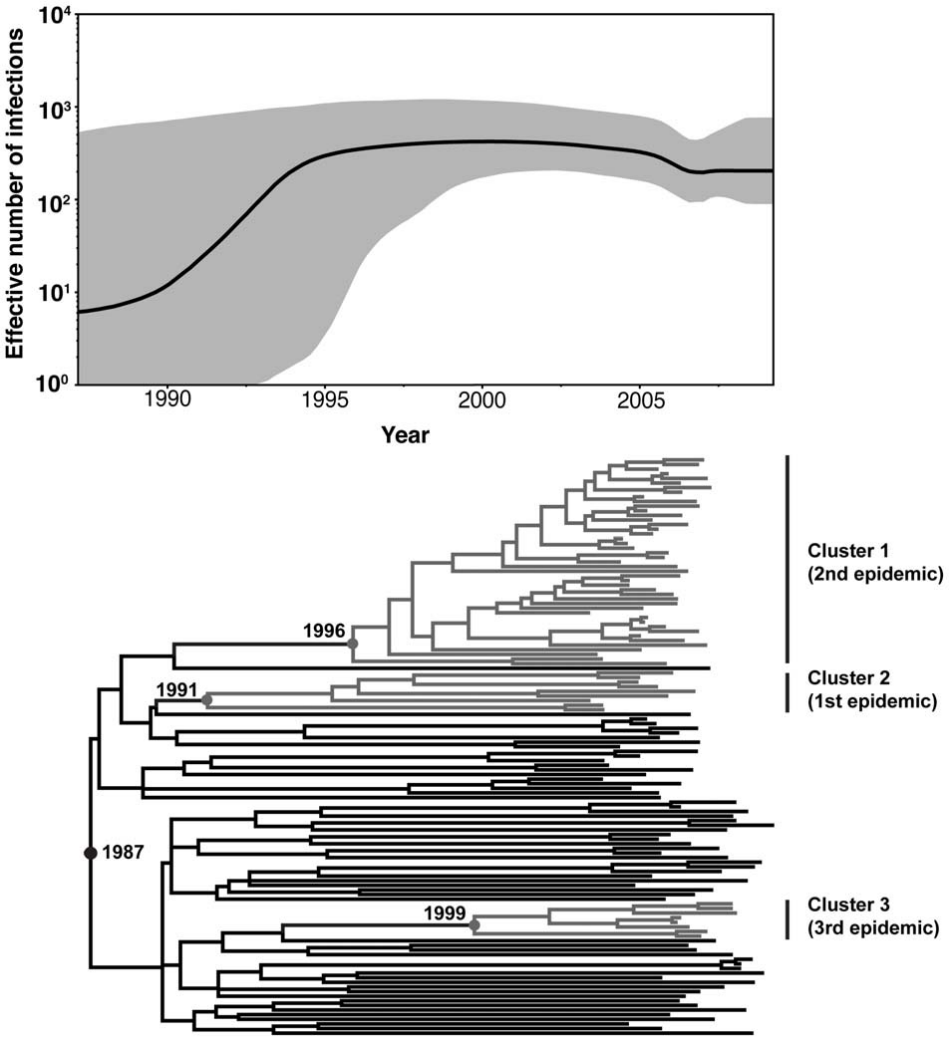
Bayesian Skyline Plot and dated phylogeny of the the Hong Kong MSM HIV-1 subtype B viruses. Nonparametric reconstruction of the epidemic history with appropiate confidence limits and time-scaled phylogenies of the *pol* gene are shown. The demographic history of the subtype B virus among Hong Kong MSM represented in black, and the 95% confidence limits of the estimate are represented in grey. The tree represents the phylogenetic relationships of the sequences has the same time scale as the skyline plot.

Regarding this substitution rate, the most recent common ancestor (tMRCA) for the HIV-1 subtype B circulating among Hong Kong MSM was estimated to be 1987. The tMRCA for the three clusters were 1996 (Cluster 1), 1991 (Cluster 2) and 1999 (Cluster 3) (Figure 2).

### Demographic history and epidemic parameter of Hong Kong subtype B MSM

Bayesian skyline plot analysis was also used to infer the demographic history of subtype B epidemics among MSM in Hong Kong (Figure 2). According to skyline plot, there was an exponential growth among the subtype B MSM during the first 10 years after the introduction of subtype B into the Hong Kong MSM population in 1987. A growth rate (*r*) at 0.78 year^−1^ (0.29–1.32), corresponding to a doubling time of 0.89 years (10.7 months) was estimated. A constant population spread was followed between the mid-1990s and mid-2000s. Starting from 2006, a more recent decline in growth rate was observed. These results suggested that the logistic population growth model was better to explain the demographic information contained in the data set than the exponential population growth model.

## Discussion

HIV-1 epidemic in the Asia Pacific region has long been concentrated in specific populations, namely sex workers and their clients, injecting drug users [1]. In recent years, the epidemic is steadily expanding into the MSM population especially in the high-income economics including Australia, Japan, Hong Kong, Taiwan and Singapore [7], [12], [13]. In Hong Kong, the continuous expansion of MSM from less than 20% in 1984 to more than 40% in 2009 among the total HIV-1 population per year in Hong Kong indicated that sex between men has become a major route of infection in our locality [5]. Also our recent studies highlighted that HIV-1 subtype B was one of the predominant genotypes circulating in Hong Kong starting from the 1980s [14], [15].

This study incorporated the use of phylogenetic and demographic data for investigating the evolution and transmission of HIV-1 subtype B in the Hong Kong MSM population. The results can help us understand more about the epidemic changes of HIV-1 in high-income Asian countries.

The Bayesian phylogenetic analysis in this study revealed the mixed phylogeny of the Hong Kong and Western subtype B isolates. The dated phylogenetic analysis allowed us to further estimate the tMRCA for the Hong Kong subtype B samples in MSM population to be in 1987, which is about 3 years after the first identified subtype B case in Hong Kong [16]. After the introduction of the subtype B virus into the local MSM population, the strains further spread in a multiple sourcing approach. According to the epidemiology background collected in our study, most of the Hong Kong local Chinese MSM caught infection inside the Hong Kong territory. On the other hand, >50% Caucasian MSM declared to catch infection in other places including the mainland China, other Southeast Asian or Western countries. This further denoted the HIV-1 subtype B epidemics among Hong Kong MSM were mainly local originated.

Moreover, the viral sequences isolated from the MSM in Hong Kong did not carry any DRM. The absence of DRM agreed with the conclusion of low prevalence of transmitted HIV-1 drug resistance in Hong Kong in our recent findings [17].

The phylogenetic analysis also revealed the occurrence of 3 separate subtype B epidemics in the local Chinese MSM population starting from the 1990s. Comparing to the previously reported subtype B transmission clusters between 2002 and 2006 [7], our study with samples collected between 2005 and 2009 revealed a larger scale of HIV-1 transmission among MSM in Hong Kong after 2005.

The first epidemic (Cluster 2) was estimated to start in 1991. This epidemic was comparatively small-scaled and our recent study showed that 3 patients of this cluster sourced their sex partners in the social cycle through visiting sauna [9]. Although the data was not statistically significant, sauna should be counted as a major transmission route of the Cluster 2 epidemic. The low median viral load and limited number of saunas in Hong Kong suppressed the rate of transmission, which may restrain this epidemic in small-scale. However, the high percentage of patients in this cluster required Highly Active Antiretroviral Therapy (HAART) treatment afterwards suggested that the virus strains circulating in the Cluster 2 epidemic may be more pathogenic and further study will be required.

Another subtype B epidemic (Cluster 1) was estimated to introduce into the Hong Kong MSM population in 1996. This epidemic was found to be a large-scale transmission and it is still expanding among the Hong Kong MSM after 13 years of transmission. Among the 45 patients in this cluster, 7 of them were confirmed sourcing sex partners through internet [9]. The common use of internet in Hong Kong can further explain the high spreading numbers for this cluster. Also, patients in this cluster demonstrated a higher median viral load and more severe clinical symptoms developed comparing to other clusters. However, due to the limited number of epidemiologically confirmed patients, further study will be necessary.

The latest subtype B epidemic (Cluster 3) identified in this study was estimated to start in 1999. This study showed that this epidemic was mainly circulating among local Chinese MSM and small number of Caucasian MSM.

For the non-clustering samples, multiple origins were identified among both Chinese and Caucasian MSM in Hong Kong. Although a higher number of CDC B1–B3 category were observed among the scattered MSM patients, we cannot conclude the virulence of the scattered HIV-1 strains due to the lack of statistical support.

On the basis of the Bayesian phylogeny and clinical epidemiology, this study revealed the HIV-1 subtype B epidemic in the Hong Kong MSM population in the past 30 years. We demonstrated that the subtype B epidemic in the MSM population grew after a logistic model, which was in line with the findings of other studies concerning the expansion of the subtype B epidemic in high-income Western countries [18]. The exponential increase of HIV-1 MSM population in early 1990s might have been contributed by the incomplete viral load suppression in using zidovudine mono-therapy before 1996, which was the introduction year of HAART. Since the use of HAART could more effectively suppress viral load and slow down the HIV-1 transmission, the infected population size between 1996 and 2005 maintained constant. The MSM population size of subtype B epidemic was slightly decline beyond 2005. Our previous study proposed that the recent introduction of CRF01_AE transmission clusters into the Hong Kong MSM population may account for the decrease in the number of people infected with HIV-1 subtype B after 2005 [19].

From our study, we observed that HIV-1 population dynamics in Asia may be different from those in the western countries, since subtype B and non-B genotypes are both prevalent among MSM patients in Asia, whereas subtype B is the most prevalent genotype in Europe and the North America [1]. The findings of this study supported by our previous study on CRF01_AE isolates in Hong Kong provided a comprehensive phylodynamics model for other Asian high-income cities, suggesting the cross influence of subtype B and non-B MSM population growth during years [19].

In conclusion, our study elucidated the evolutionary and demographic history of HIV-1 subtype B among the Hong Kong MSM population. The understanding of transmission and growth model of the subtype B MSM pandemic in Hong Kong provides more information on the HIV-1 transmission among MSM population in other Asian high-income countries. Further studies are necessary to reveal the demographic history of other genotype viruses that are commonly found in Asia.

## Materials and Methods

Ethics approval has been obtained from the Institutional Review Board of the University of Hong Kong/Hospital Authority (Hong Kong West Cluster) with Reference number UW08-070. Written inform consents were collected from all participants in this study and the ethics committee approved this consent procedure.

### Sampling and HIV-1 Genotyping

This retrospective study included a total of 125 HIV-1 subtype B *pol* gene sequences which were isolated from the first available treatment naïve plasma samples of 125 HIV-1 infected MSM patients with method as previously described [20]. They visited the Integrated Treatment Centre, Department of Health for routine genotyping resistance testing between March 2005 and April 2009. The patient epidemiological information including age, gender, ethnicity, place of birth, route of infection, viral load and CD4 cell count were collected from the Integrated Treatment Centre, Department of Health.

The protease (PR) and partial reverse transcriptase (RT) of the HIV-1 *pol* gene (1125 base pairs) were prepared by an in-house genotyping method described previously or the ViroSeq HIV-1 Genotyping System [20]. The presence of DRM was accessed by the Stanford HIV drug resistance database (http://hivdb.stanford.edu). The genotypes of the *pol* sequences were further confirmed by using the REGA HIV-1 Genotyping Tool version 2.0 [21].

### Phylogenetic reconstruction

Phylogenetic analyses for the estimation of transmission and divergence of HIV-1 subtype B clusters among MSM was performed in a single dataset consist of 125 sequences. The alignment of the 125 local subtype B PR/RT sequences plus 38 HIV-1 subtype B reference sequences isolated from Europe, USA and Australia and 2 subtype C outgroup sequences was performed using MUSCLE [22]. The best-fit nucleotide substitution model for the sequences was estimated by using MrModeltest version 2.3 [23]. Bayesian phylogenetic trees were then constructed using MrBayes under the general time-reversible (GTR) model of nucleotide substitution with gamma-distributed rate variation (Γ) and a proportion of invariable sites (*Ι*) [24]. A Markov Chain Monte Carlo (MCMC) search was made for 1×10^7^ generations using tree sampling every 1000^th^ generation and a burn-in fraction of 50%. Tree clades with posterior probability of 1 and more than 5 members were considered as epidemiological clusters.

### Evolutionary rate and divergence time of transmission cluster among MSM

The tMRCA, evolutionary rates and population growth of HIV-1 subtype B viruses among MSM in Hong Kong were estimated by using the Bayesian MCMC approach with the GTR+Γ+*Ι* substitution model as implemented in BEAST version 1.6.1 [25]. The posterior distribution, previously estimated from an independent data set of 106 subtype B *pol* sequences sampled between 1983 and 2000 in Amsterdam [11], was subsequently used as an empirical prior distribution in the coalescent analyses. For the coalescent analyses, different parametric demographic models (constant population size, exponential and logistic growth) and a nonparametric Bayesian skyline plot were compared under strict and relaxed clock conditions, and the best model was selected by means of Bayes factor using maximum likelihoods. [26]. The strength of evidence for the best model was calculated by using the Kass and Raftery method (2lnBF  =  <2, no evidence;  = 2–6, weak evidence;  = 6–10, strong evidence;  =  >10, very strong evidence) [27].

Two runs of four chains each were run for 50×10^6^ generations, with a burn-in of 5×10^6^ generations. Samples of trees and parameter estimates were collected every 100 steps and they were estimated from an effective sample size >200. The Bayesian MCMC results were analyzed by TRACER, version 1.5 (http://tree.bio.ed.ac.uk/software/tracer). Mean evolutionary rates and divergence times were calculated using TreeAnnotator version 1.6.1 after the removal of 10% burn-in.
